# Diplomatic Collaborative Solutions to Assure the Adoption of the European Single Market Amid the COVID-19 Pandemic

**DOI:** 10.3389/fpubh.2021.662170

**Published:** 2021-07-13

**Authors:** Silvia Logar, Rizzardo Alessandro

**Affiliations:** ^1^School of Social Sciences, Humanities & Law, Teesside University, Middlesbrough, United Kingdom; ^2^John XXIII Health Authority, Monastier, Vittorio Veneto, Italy

**Keywords:** COVID-19, international cooperation, health emergency and disaster risk management, borders, european union

## Abstract

As the COVID-19 crisis has shown, the lack of harmonized and coordinated actions superseding national borders represented a limit to the full implementation of already existing legal binding instruments at the European level. It is recognized that the existing levels of globalization have contributed to accelerate the large-scale transmission of viruses and increased the likelihood of a pandemic public health crisis. This article aims to highlight the importance of greater bilateral cooperation to mitigate the health and economic impact of the coronavirus disease 2019 (COVID-19) pandemic. It focuses on the implementation of diplomatic collaborative systems to assure the full implementation of the European single market as well as the adoption of standardized health information platforms as a part of pandemic preparedness and control measures.

On 31 December, 2019, a cluster of pneumonia cases of unknown etiology was recorded in Wuhan, Hubei Province, China. In January 2020, the Chinese Centre for Communicable Disease (CDC) reported a novel coronavirus as the causative agent of this outbreak, coronavirus disease 2019 (COVID-19). By then, more than 170 million cases and over 4 million deaths had been recorded worldwide (1).

As the COVID-19 crisis has shown, the lack of harmonized and coordinated actions superseding national borders represented a limit to the full implementation of already existing legal binding instruments at the European level [e.g., the European Union (EU) decision on serious cross-border threats to health (2)], including the large scale data sharing during the COVID-19 emergency (3).

In general, all member states were initially inward-looking (e.g., unilateral closure of borders), while in the latest pandemic phases a European level approach, with a clear focus on lockdown exits and economic recovery, has been adopted. The EU measures established include, among others, the following: (i) a joint bid to improve the procurement of personal protective equipment and vaccines; (ii) increased funding for pharmaceutical research; (iii) relaxed regulatory enforcement; and (iv) interventions to support the free circulation of goods in the single market, reducing border backlogs. A list of fiscal rules has been proposed to address the unprecedented deficit spending (e.g., pandemic emergency purchase programme announced by the European Central Bank) (4).

However, while the economic growth potential generated through European integration is one of the major assets of the EU membership, the cooperation policies during the COVID-19 emergency remained very much in the hands of member states.

The bilateral cooperation has, indeed, played a critical role in guaranteeing the four freedoms of the European single market (referring to the EU as one territory without any internal borders or other regulatory obstacles to the free movement of goods, capital, services, and people) during the pandemic; this resulted, however, in fragmented governance of the EU borders (5).

However, for the best part of 2020, the reestablishment of internal border controls and the introduction of restrictions or outright bans on travel in response to the COVID-19 pandemic have severely affected the rights of citizens of the EU to move freely. The disintegration of free movement was particularly well-illustrated in the case of Romanian seasonal workers, around 250,000 of whom in March 2020 had to return from EU member states (namely, Italy, France, Spain, Austria, and Germany) to Romania after their employment ended. In this case, the termination of employment contracts, whose legality remains uncertain, affected the rights of the workers to reside on the territory (6). Romanian truck drivers and road travelers got blocked in Austria and Hungary, as a result of national quarantine or lockdown measures, thus requiring the intervention of Romanian authorities to negotiate bilateral solutions with their EU counterparts (7).

Mobile and seasonal workers are often essential for the member states, for example in sectors such as healthcare, care for the elderly or persons with disabilities, or in construction (more than 8% of mobile workers are employed in the healthcare and social work sectors). The COVID-19 pandemic revealed numerous structural shortcomings in the bilateral regulatory frameworks. The major challenge is, currently, the lack of harmonized interpretation of EU law by member states, such as the recently revised Posted Workers Directive, which leads, among others, to a lack of legal clarity and bureaucratic burdens for companies providing services in more than one member state (8).

In March 2021, the French government tightened controls at the German (Moselle region) border to slow down the infection rate (particularly after the detection of the South African strain of the virus). France and Germany have agreed that mobile workers (~16,000) will have to have proof of a negative COVID-19 antigen test in the previous 48 h. French cross-border workers with Luxembourg have not been targeted by the new PCR test requirement (9).

The Slovenian and Switzerland governments are offering free antigenic tests for Italian mobile workers (on a weekly basis); Italy introduced an exception to quarantine and PCR test requirements for the mobile and seasonal workers coming from other EU Countries (10, 11).

The Belgian government has put in place different systems for frontier workers traveling to and from Belgium.

If a person is working in a vital sector and/or an essential profession (e.g., healthcare), a special certificate can be obtained online.

Employers of frontier workers who need to cross the Belgian-Luxembourg border need to complete, sign, and stamp a certificate provided by the Luxemburg authorities, whereas, frontier workers living in France and working in Belgium need three documents to cross the border [(i) the attestation from the French employer, (ii) the French personal attestation, and (iii) the Belgian attestation]. Similarly, frontier workers living in Germany and working in Belgium can cross the border into Belgium with an attestation signed and stamped by their employer (12).

Member states are also required to ensure the seamless free movement of goods across the single market. On 28 October, 2020, the commission proposed the adoption of the Green Lane approach: border crossings are open to all freight vehicles carrying goods where any checks or health screenings should not take more than 15 min (including rail, waterborne freight, and air cargo).

The Green Lane implementation is the result of bilateral agreements among member states, leading to huge gaps in its practical and full achievement (13).

For instance, the estimated border crossing time from Bulgaria to Romania is 45 min, while it takes ~2 h on the opposite way. The waiting time from Italy to Austria is 10 min, but trucks coming from the reverse direction take 45 min to enter. Poland to Belarus border crossing time is 4 h both ways (14).

The Italian Friuli Venezia Giulia (FVG) region and Slovenia, a neighboring country with comparable socio-economic features, implemented, so far, a successful cooperation plan for international epidemic control, including, among others, improved governance coordination as well as timely and adequate information and knowledge sharing.

During the lockdown period in Italy, which took place from 11 March, 2020 to 3 June, 2020, from the border of FVG, it was possible to enter Slovenia at one out of six control points with border officers from both countries collaborating in its management. While nobody was allowed to move in or out of confines, the corridor assured cross-border access for work-related reasons, emergencies, or health issues (15).

After an intense diplomatic dialogue, Italian and Slovene authorities defined a special corridor across Gorizia (Italy) and Nova Gorica (Slovenia) to replace fluid, busy borders for trades, after which 450 vehicles, mostly directed to Poland and Ukraine, have been prohibited to cross Slovenia and proceed to the destination, as well as to turn back to Italy, have been stuck for days at the border. The agreement assured the delivery of essential goods, such as food and medical items, especially for the countries that were the most dependent on imports to cover basic needs (16).

However, this scheme may need augmentation under a framework of improving existing organizational, financial, and legal uncertainties.

In terms of health information systems, member states, with the support of the commission, have agreed on a set of technical specifications to ensure a safe exchange of information among national contact tracing apps based on a decentralized architecture (17). This includes the proposal to establish a European Digital Green Certificate to facilitate the safe and free movement of citizens within the EU during the COVID-19 pandemic. However, the extent of its applicability remains unsure, as the national authorities are in charge of issuing the certificate (18).

This is further reflected in the fact that while the member states successfully shared relevant data, in many circumstances, the level of quality and detail varied significantly. For example, the number of cases by age and sex has not been provided, thus limiting the stratified trends of the pandemics. Another limitation is that variation in the quality and completeness of national surveillance data impaired the quality of international surveillance. Key information such as the criteria adopted for testing, which has a direct effect on the number of confirmed cases and deaths reported, has not been fully shared, fostering also a lack of trust among member states (19). All of these factors can limit the ability of the cross-border countries to learn in real-time from available data, and potentially undermined the ability of the countries to timely respond to the pandemic.

## Conclusion

The coronavirus pandemic which started in 2019 and is still ongoing has demonstrated the need for bilateral cooperation for the effective mitigation of cross-border threats, including health crises.

International cohesion is critical to assure successful containment of the pandemics and maintaining trust between governments in fast-moving times; a similar level of resources allocated to both countries is essential for a coordinated approach (12).

This pandemic urges all member states to enhance bilateral cooperation and calls for political support and commitment of resources to achieve a more coordinated cross-border approach to control the disease (including acute response).

## Data Availability Statement

Publicly available datasets were analyzed in this study. This data can be found here: https://www.regione.fvg.it/rafvg/comunicati/homepage.act?dir=/rafvg/cms/RAFVG/notiziedallagiunta/.

## Author Contributions

All authors listed have made a substantial, direct and intellectual contribution to the work, and approved it for publication.

## Conflict of Interest

The authors declare that the research was conducted in the absence of any commercial or financial relationships that could be construed as a potential conflict of interest.
